# How do Adverse Childhood Experiences get Under the Skin to Promote Cardiovascular Disease? A Focus on Vascular Health

**DOI:** 10.1093/function/zqac032

**Published:** 2022-06-15

**Authors:** Nathaniel D M Jenkins, Austin T Robinson

**Affiliations:** Integrative Laboratory of Applied Physiology and Lifestyle Medicine, Department of Health and Human Physiology, University of Iowa, Iowa City, IA 52242, USA; Abboud Cardiovascular Research Center, Carver College of Medicine, University of Iowa, Iowa City, IA 52242, USA; Neurovascular Physiology Laboratory, School of Kinesiology, Auburn University, Auburn, AL 36849, USA

**Keywords:** early life stress, psychosocial stress, social determinants of health, vascular dysfunction, flow mediated dilation, cerebral blood flow

## A Perspective on “The Link Between Childhood Adversity and Cardiovascular Disease Risk: Role of Cerebral and Systemic Vasculature"

Adverse childhood experiences (ACEs) represent stressful, and potentially traumatic, events that include abuse, neglect, and household dysfunction. The landmark Center for Disease Control-Kaiser Permanente ACE study revealed that ACE exposure associated in a dose-dependent manner with the incidence of cardiovascular diseases (CVDs) such as ischemic heart disease and stroke.^1^ Notably, a 2017 systematic review indicated that 92% of the studies included reported an association between ACEs and CVDs.^2^ Alarmingly, 60% of individuals in the US report exposure to at least 1 ACE, and ∼25% report exposure to ≥3. Despite the pervasiveness of ACEs and robust evidence supporting the association between ACEs and CVD, our current understanding of how ACEs promote CVD is limited. In this issue of *Function*, Rodriguez-Miguelez and colleagues^3^ provide important evidence to further reveal how ACEs ‘get under the skin’ to promote CVD.

Initial evidence regarding the pathophysiological mechanisms by which ACEs promote CVD largely emerged from pre-clinical, mouse models of early life stress, such as maternal separation with early weaning^4^. Recently, Jenkins et al.^5^ translated the preclinical evidence on ACEs and vascular function to young adults. Specifically, young adult women (∼20 years old) with moderate-to-severe (≥4) ACE exposure exhibited endothelial dysfunction, as measured by the flow mediated-dilation (FMD) technique. Rodriguez-Miguelez and colleagues^3^ confirmed the finding of impaired FMD in a cohort of young adult men and women (∼32 years old) and extended these findings by performing comprehensive assessments of medial prefrontal cortex (mPFC) blood flow, microvascular function, and arterial stiffness.

The authors used near-infrared spectroscopy to non-invasively monitor cerebral hemodynamics in the mPFC, which exerts top-down control over the amygdala. Chronic stress dysregulates PFC control over the amygdala, promoting increased amygdalar activity,^6^ which is associated with CVD events.^7^ In the present study, the ACEs group demonstrated reduced mPFC blood flow. Thus, it is plausible that ACEs cause aberrant mPFC control of amygdalar activity which subsequently leads to altered autonomic function and vascular dysfunction promoting the development of incident CVD. Rodriguez-Miguelez et al. also used laser doppler flowmetry to quantify cutaneous (i.e., skin) microvascular responses to multiple vasodilatory stimuli. They demonstrated that individuals with ACEs exhibit microvascular dysfunction, which is an antecedent to atherosclerosis. While a previous report demonstrated ACE exposure was associated with peripheral arterial stiffening,^8^ these results had not yet been extended to central elastic arteries (e.g., the aorta). In the present study, individuals with ACEs exhibited central arterial stiffening as indicated by carotid-femoral pulse wave velocity (cf-PWV). Individuals with ACEs had cf-PWVs that were ∼1 m/s greater than the control group. Importantly, a 1 m/s increase in cf-PWV is associated with ∼15% higher risk for CVD mortality.

The findings in the current article suggest an important link between ACEs and vascular dysfunction despite no differences between the groups in traditional risk factors such as blood pressure or circulating lipids, glucose, and c-reactive protein. Similarly, Jenkins et al. also found that vascular dysfunction was present in those with ACEs without deviations in traditional risk factors.^5^ While ACEs are associated with increased likelihood of risky health behaviors, it appears that the association between ACEs and CVD may be independent of certain risky behaviors. For example, Jenkins et al.^5^ excluded individuals who smoked or used illicit drugs, and the ACEs cohort was matched to the control group for physical activity status. The Georgia Heart and Stress Study demonstrated an association between ACE exposure and increased systolic and diastolic blood pressure (that becomes apparent in late young adulthood (34 – 38 years)) that persisted after adjustment for physical activity, smoking, and illicit drug use.^9^ These previous findings imply that ACEs promote vascular dysfunction and hypertension risk in a manner that is largely independent of specific risky health behaviors. However, the interaction between ACEs, social determinants of health, and health behaviors remain important areas for future research to further clarify the association between ACEs and CVDs.

Potential future directions of ACEs and cardiovascular health research include examining whether sleep, an important health behavior that influences vascular function, may mediate the effects of ACEs on vascular function. Using a lifespan approach to examine how the neighborhood context (educational opportunities, pollution, crime) in childhood shapes exposure to, and consequences of, ACEs that translate into health disparities in young adulthood would also be informative. There is also a lack of data on the importance of specific childhood developmental periods in which ACEs occur and how they may have differential effects on future vascular dysfunction (e.g., ACEs experienced as a toddler vs. adolescence). From a mechanistic standpoint, it remains unclear how ACEs may elicit vascular dysfunction in humans. However, data in mice^4^ and young adults^5^ support a potential role of oxidative stress. Future studies using acute antioxidant and/or anti-inflammatory supplementation would provide additional insight. Lastly, the finding of lower mPFC blood flow in individuals with ACEs have important implications that also spark several potential avenues for future examination. For example, it has been reported that ACE exposure is negatively associated with inhibition ability, cognitive flexibility, and working memory in young adults^10^ – executive functions that are attributed to the PFC. These findings highlight that ACEs may promote impairments in cognitive function and altered cerebrovascular blood flow which are important risk factors for cognitive decline and dementia in aging adults.

In summary, while the primordial prevention of ACEs would be ideal, the sheer number of individuals exposed to ACEs worldwide makes this goal unlikely. The pervasiveness of ACEs in our society emphasizes the dire need to unravel the pathophysiological and bio-behavioral mechanisms by which ACEs promote vascular dysfunction in young adults and eventually CVDs with further aging. Importantly, the data presented by Rodriguez-Miguelez and colleagues indicate that a similar dose-response relationship between ACE exposure and impaired function is present across various vascular beds using multiple vascular assessment methodologies – a particularly notable and robust finding. Together with pre-clinical evidence^4^ and previous data in young adults,^5^ there seems to be little doubt that ACE exposure promotes vascular dysfunction. It also seems that vascular dysfunction may be one of the earliest observable pathophysiological mechanisms on the pathway between ACE exposure and overt CVD. The question now is, what are the mechanisms and are they targetable?

## Data Availability Statement

No new data were generated or analyzed in support of this research.
